# Supplementary figures and images for: A decavalent composite mRNA vaccine against both influenza and COVID-19
Source: mBio. 2024 Aug 6;15(9):e00668-24. doi: 10.1128/mbio.00668-24 (PMC11389412; doi:10.1128/mbio.00668-24)

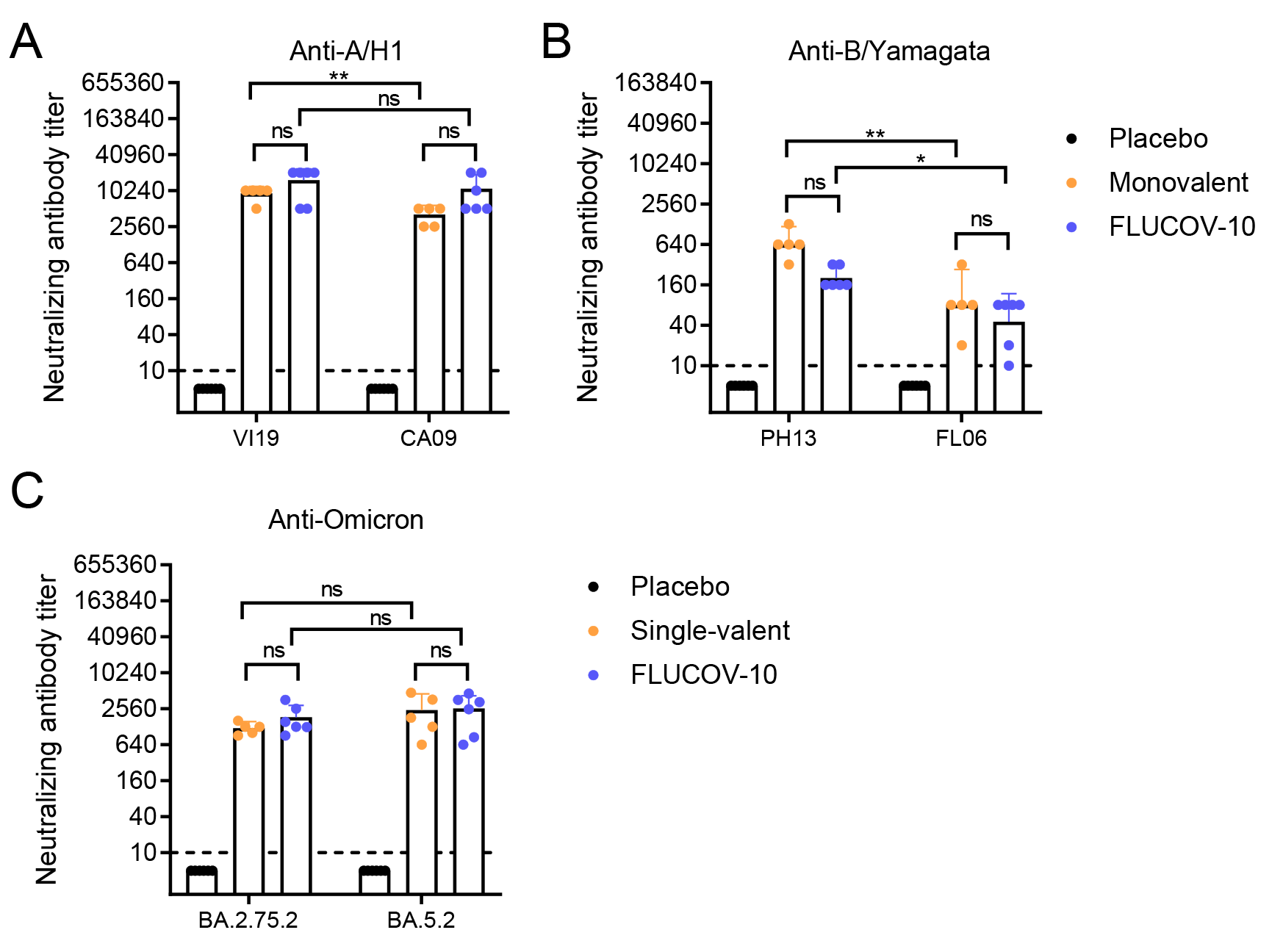

Supplement: Figure S1 — FLUCOV-10 immunization elicits a cross-reactive humoral immune response in BALB/c mice (related to Fig. 2). [file mbio.00668-24-s0001.tif]

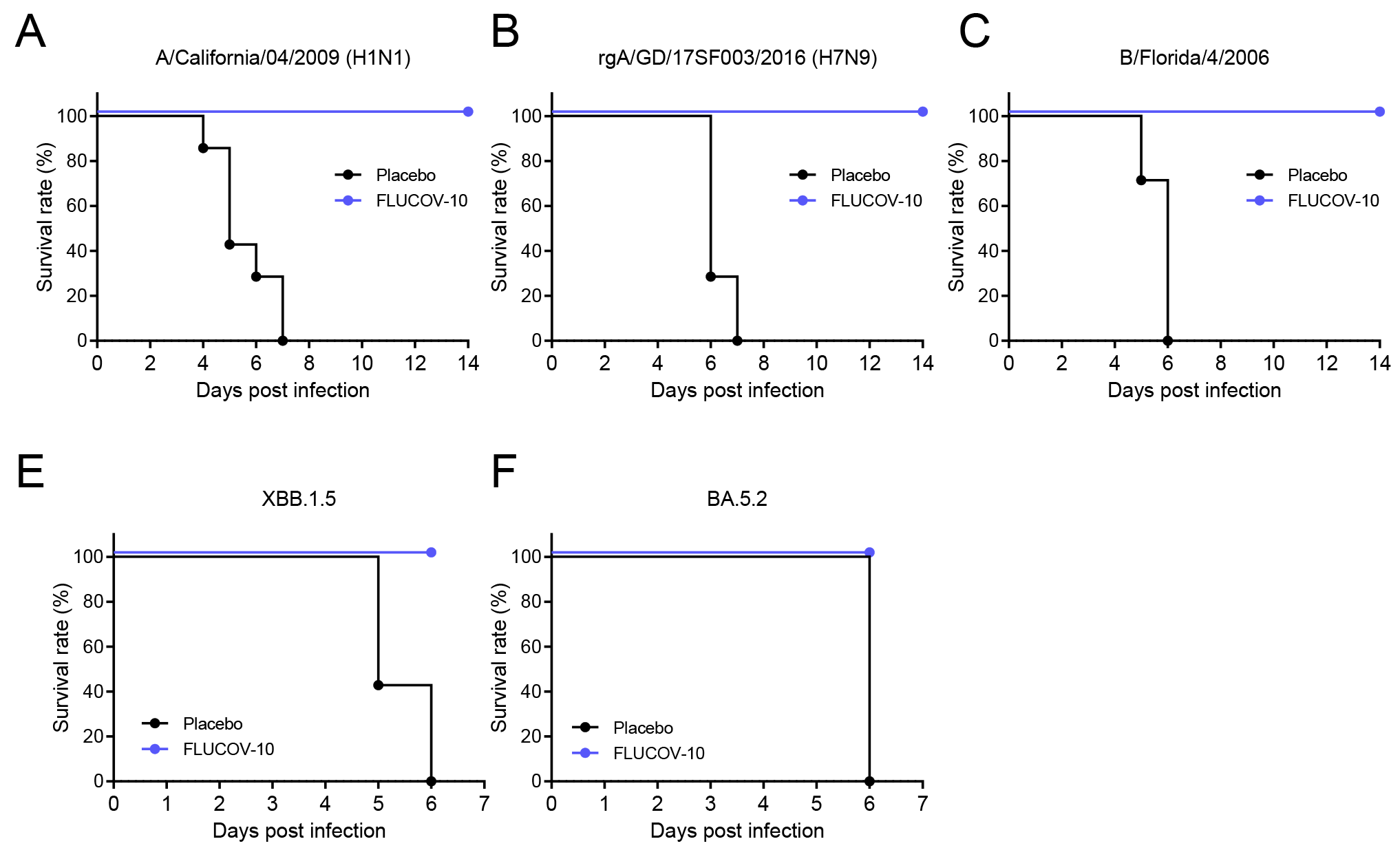

Supplement: Figure S2 — Survival rates in mice immunized with FLUCOV-10 and challenged with influenza or SARS-CoV-2 viruses (related to Fig. 4 and 5). [file mbio.00668-24-s0002.tif]

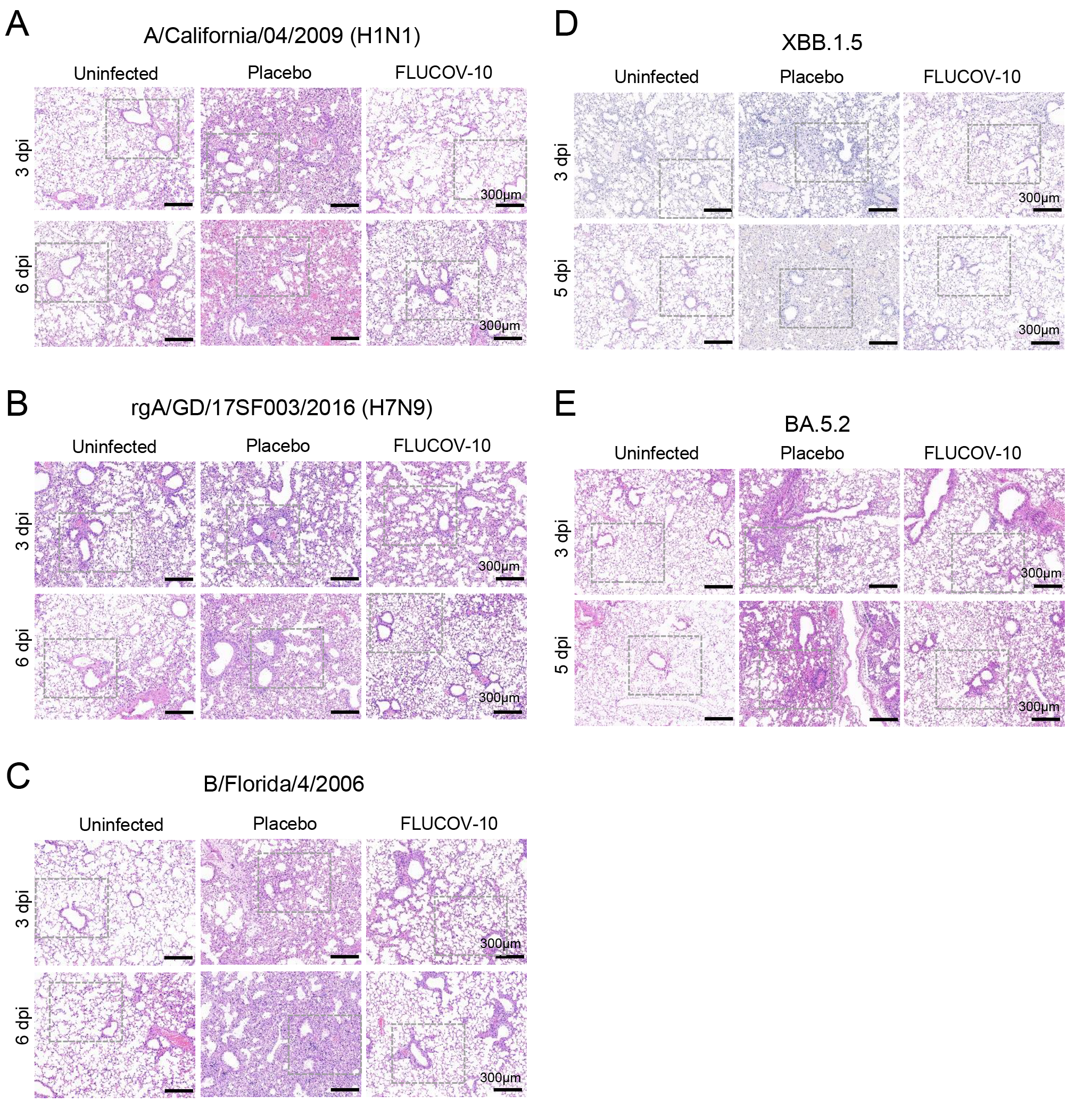

Supplement: Figure S3 — Lung lesions in uninfected and infected mice (related to Fig. 4 and 5). [file mbio.00668-24-s0003.tif]
