# Supplementary material for: A decavalent composite mRNA vaccine against both influenza and COVID-19
Source: mBio. 2024 Aug 6;15(9):e00668-24. doi: 10.1128/mbio.00668-24 (PMC11389412; doi:10.1128/mbio.00668-24)
Supplement: Legends — Supplemental figure legends. [file mbio.00668-24-s0004.docx]

**A decavalent composite mRNA vaccine against both influenza and COVID-19**

Yang Wang^1,2,#^, Qinhai Ma^1,#^, Man Li^3,#^, Qianyi Mai^1^, Lin Ma^4^, Hong Zhang^3^, Huiling Zhong^3^, Kailin Mai^1^, Nan Cheng^4^, Pei Feng^5,6^, Peikun Guan^2^, Shengzhen Wu^1^, Lu Zhang^7^, Jun Dai^1,2,7,*^, Biliang Zhang^3,8,*^, Weiqi Pan^1,5,*^, Zifeng Yang^1,2,5,6,*^

^1^State Key Laboratory of Respiratory Disease, National Clinical Research Center for Respiratory Disease, Guangzhou Institute of Respiratory Health, The First Affiliated Hospital of Guangzhou Medical University, Guangzhou, China;

^2^Guangzhou National Laboratory, Guangzhou, China;

^3^Argorna Pharmaceuticals Co., Ltd., Guangzhou, China;

^4^Guangzhou RiboBio Co., Ltd, Guangzhou, China;

^5^Respiratory Disease AI Laboratory on Epidemic and Medical Big Data Instrument Applications, Faculty of Innovation Engineering, Macau University of Science and Technology, Macau SAR, China;

^6^State Key Laboratory of Quality Research in Chinese Medicine, Macau Institute for Applied Research in Medicine and Health, Macau University of Science and Technology, Taipa, Macau SAR, China;

^7^Technology Centre, Guangzhou Customs, Guangzhou, China;

^8^State Key Laboratory of Respiratory Disease, Laboratory of Computational Biomedicine, Guangzhou Institutes of Biomedicine and Health, Chinese Academy of Sciences, Guangzhou, China;

^#^ These authors contributed equally to this work.

* Correspondence: jeffyah@163.com (Z.Y.); panweiqi@gird.cn (W.P.); bill.zhang@ribobio.com (B.Z.); 19915302@qq.com (J.D.)

**Supplementary Information**

**Supplemental Figure 1. FLUCOV-10 immunization elicits a cross-reactive humoral immune response in BALB/c mice (related to Figure 2).**

A. BALB/c mice were vaccinated i.m. with the FLUCOV-10, monovalent A/H1 mRNA vaccines derived from FLUCOV-10 or a placebo. Neutralizing antibody titers against vaccine matched (A/Victoria/2570/2019, VI19) and antigenically distinct (A/California/04/2009, CA09) A/H1N1pdm09 influenza viruses were determined 14 days post second immunization by micro-neutralization assays. B. BALB/c mice were vaccinated i.m. with the FLUCOV-10, monovalent B/Yamagata mRNA vaccines derived from FLUCOV-10 or a placebo. Neutralizing antibody titers against vaccine matched (B/Phuket/3073/2013, PH13) and antigenically distinct (B/Florida/4/2006, FL06) B/Yamagata influenza viruses were determined 14 days post second immunization by micro-neutralization assays. Data are presented as geometric means ± 95% CI (n = 5 or 6). C. BALB/c mice were vaccinated i.m. with the FLUCOV-10, monovalent BA.2.75.2 mRNA vaccines derived from FLUCOV-10 or a placebo. Neutralizing antibody titers against vaccine matched (BA.2.75.2) and antigenically distinct (BA.5.2) SARS-CoV-2 viruses were determined 14 days post second immunization by micro-neutralization assays. ns, non-significant; *, *p* < 0.05; **, *p* < 0.01.

**Supplemental Figure 2. Survival Rates in Mice Immunized with FLUCOV-10 and Challenged with Influenza or SARS-CoV-2 Viruses (related to Figures 4 and 5).**

The mice were challenged 3 weeks post second immunization with indicated viruses and the survival rates were monitored for 7 or 14 days.

**Supplemental Figure 3: Lung pathology in Uninfected and Infected Mice (Related to Figures 4 and 5).** BALB/c mice received two doses of either FLUCOV-10 or placebo. Two weeks post the final dose, mice were challenged with influenza strains A/California/04/2009 (H1N1) (A), rgA/GD/17SF003/2016 (H7N9) (B), or B/Florida/4/2006 (C). K18-hACE2 mice were administered two doses of FLUCOV-10 or placebo and subsequently infected with SARS-CoV-2 variants XBB.1.5 (D) or BA.5.2 (E). Hematoxylin and eosin (H&E) stained lung tissues from both infected and uninfected mice are presented. Dashed boxes in this figure indicate the areas corresponding to the H&E images discussed in Figures 4 and 5.
